# Rollout of a care bundle for intracerebral haemorrhage across the North of England: a qualitative study

**DOI:** 10.1136/bmjoq-2025-004099

**Published:** 2026-08-26

**Authors:** Lisa Brunton, Paul Wilson, Adrian R Parry-Jones

**Affiliations:** 1 Division of Population Health, Health Services Research & Primary Care, The University of Manchester Faculty of Biology Medicine and Health, Manchester, UK; 2 Division of Cardiovascular Sciences, The University of Manchester Faculty of Biology Medicine and Health, Manchester, UK; 3 Geoffrey Jefferson Brain Research Centre, Northern Care Alliance NHS Foundation Trust Salford Care Organisation, Salford, UK

**Keywords:** Implementation science, Qualitative research, Evaluation methodology

## Abstract

**Background:**

A care bundle was developed and implemented at a hospital in North West England to improve the management of intracerebral haemorrhage (ICH) and was associated with a 44% reduction in 30-day case fatality. It was scaled out to the other hyperacute stroke units (HASUs) in the region. Findings showed the bundle could be successfully implemented into new settings, but challenges impeded its implementation. The findings were used to develop an implementation strategy to support roll-out outside the region. The ABC-ICH care bundle was then scaled up as a quality improvement initiative across six regions in the North of England. This paper reports on the qualitative process evaluation that was conducted in parallel.

**Methods:**

Multiple qualitative methods (45 interviews, six meeting observations and a document analysis) were used to capture how the bundle was implemented in HASUs across the six regions. We used the integrated-Promoting Action on Research Implementation in Health Services (i-PARIHS) framework to theoretically inform the study.

**Findings:**

Key facilitators in the uptake of the bundle were the clarity of the intervention, its compatibility with clinicians’ values, its perceived relative advantage compared with current ICH care and the ability to locally tailor protocols. Key barriers to implementing the care pathway (judicious referral to neurosurgery) included lack of recipients’ skills and confidence and prevailing organisational cultural norms which meant that most clinicians continued to refer all patients regardless of whether they met eligibility criteria. Lack of routine monitoring of process targets by local implementation teams impeded use of plan-do-study-act cycles to further improve care. Local contextual issues impeded or enabled effective facilitation.

**Conclusion:**

The findings recognise the importance of understanding what happens when the bundle is introduced into new settings outside of its local context and identifies lessons for future national roll-out.

**WHAT IS ALREADY KNOWN ON THIS TOPIC**

**WHAT THIS STUDY ADDS**

- When the ABC-ICH care bundle was scaled up to a further six regions across the North of England, we found that a key facilitator to adoption was its fit with clinicians’ values and perceived advantage over current care.
- Clinicians’ lack of skills and confidence meant they continued to refer all patients to neurosurgery despite a care pathway being in place to refer only eligible patients. Contextual and facilitation issues meant that few sites routinely monitored process targets which limited their ability to further improve ICH care.

**HOW THIS STUDY MIGHT AFFECT RESEARCH, PRACTICE OR POLICY**

- This study shows that the ABC-ICH care bundle can be scaled up to settings outside of where it was developed.
- We identified improvements to make to the implementation strategy before national roll-out, including the need to strengthen monitoring and feedback mechanisms, collaborate with local networks to coordinate regional efforts, limit the number of implementation roles clinicians take on to reduce burden and provide targeted staff training in blood volume calculation to support pathway adherence.

## Introduction

 Intracerebral haemorrhage (ICH) accounts for only 13% of all strokes in the UK^1^ but is associated with disproportionately worse outcomes for mortality and disability compared with other stroke subtypes. Approximately 40% of ICH patients die within 30 days of onset and most survivors experience some form of disability,^2^ which places burden on healthcare systems and individuals alike.

Studies suggest that therapeutic nihilism can limit the clinical management of ICH, leading to poorer outcomes. A study from the UK, undertaking a retrospective secondary analysis of a UK national quality register, found that ICH patients were significantly more likely to be started on palliative care within 72 hours of admission, compared with patients with acute ischaemic stroke, independent of their age, consciousness level and premorbid health and this was shown to be independently associated with an increased risk of death.^3^ A recent multicentre observational retrospective study in the USA found that time to treatment for patients with ICH was significantly longer than for patients with acute ischaemic stroke and was associated with higher mortality rates and worse discharge outcomes.^4^ However, recent randomised clinical trials show that early intervention of blood pressure (BP) management and timely anticoagulant reversal in ICH patients can lead to better functional outcomes (modified Rankin Scale) at 3–6 months compared with usual care.^5 6^ This indicates a need to deliver quicker, evidence-based treatment to improve outcomes for ICH patients.

To improve the management of ICH care, the ABC-ICH care bundle (see box 1) was developed to guide clinicians to deliver evidence-based interventions recommended in national guidelines.^7 8^ In 2015–2016, a quality improvement project showed that effective delivery of the ABC-ICH care bundle at a large hyperacute stroke unit (HASU) in Greater Manchester, UK, was associated with a 44% reduction in 30-day case fatality.^9^ A reduction in early (<24 hours) Do Not Resuscitate (DNR) orders was also observed as an indirect consequence of implementing the ABC-ICH care bundle and attributed to clinicians taking a more optimistic approach to treating ICH patients.^10^

Box 1 **Components of ABC-ICH care bundle**

The ABC-ICH care bundle comprises the following process targets:

- Rapid **a**nticoagulant reversal for eligible individuals with a door-to-needle time of <120 min.
- Delivery of intensive **b**lood pressure lowering for individuals arriving within 6 hours of stroke onset and with a systolic blood pressure on arrival >150 mm Hg, with a door-to-target time of <120 min of arrival.
- A **c**are pathway for prompt and appropriate referral to neurosurgery.

Implementation of the ABC-ICH care bundle was scaled up to the two other HASUs within Greater Manchester from April 2017. While no significant change to 30-day mortality was observed in either site, a mixed-methods evaluation found that one HASU significantly reduced median anticoagulant reversal door-to-needle time from 132 min (IQR: 117–342) to 76 min (IQR: 64–113.5) and BP lowering door-to-target time from 345 min (IQR: 204–866) to 84 min (IQR: 60–117). No significant improvements were seen at the other HASU.^11^ The qualitative findings explained these differences, highlighting effective internal facilitation at the successful site (early identification of project team with well-defined roles, stakeholder engagement, well-attended launch event and closely monitoring data with debriefs after each ICH patient) versus the other site, where staff changes hindered ownership of local implementation. Context also played a role: the successful HASU had a small, dedicated stroke nursing team who drove bundle delivery, and had a dedicated three-bedded HASU bay within their emergency department (ED), while the other HASU contended with staff shortages throughout the duration of the improvement project. Both HASUs recognised the need for ongoing implementation support due to staff turnover. These insights informed improvements for future roll-out.^11^

### The North of England scale-up

Following the early scale-up to two additional HASUs in Greater Manchester, the intervention was selected for broader expansion across six regions in the North of England, as part of a strategy to improve ICH care. This offered the opportunity to assess the effectiveness of the ABC-ICH care bundle at scale and to understand the processes that enabled or challenged its implementation across different contextual settings.

A phased scale-up of the ABC-ICH care bundle quality improvement programme across the North of England was planned, with each region to be allocated a staggered bundle launch at 1-month intervals. Six regional groups were established, each centred around their neurosurgical unit and their referring HASUs. The scale-up was coordinated by a central project team in Manchester. Each regional group was to be supported by a regional project lead and a neurosurgical lead. Within each HASU, an implementation team would be established, comprising a lead consultant, a lead nurse and a data lead. Roles and responsibilities for each team member were defined by the central project team (see online supplemental information 1). Regional meetings were planned to be scheduled at three time points: 3 months prior to the bundle launch and then at 3 months and 6 months post launch. HASU local implementation teams were encouraged to participate in weekly or fortnightly short meetings (less than 30 min) during the project period. To aid delivery of the bundle, an app and dashboard were developed. If consistently used, the app and dashboard would capture data automatically and create automated run charts for individual HASUs to select and test changes to improve performance (plan, do, study, act (PDSA) cycles).

The outcomes of the ABC-ICH care bundle scale-up across HASUs within six regions in the North of England are reported in detail elsewhere (McManus *et al* 2025 - unpublished). In summary, patients at ABC-ICH implementation sites were significantly more likely to receive anticoagulant reversal therapy and were more likely to receive the optimal reversal agent compared with patients at non-ABC-ICH sites. However, there was no statistically significant difference in the time to administration of reversal therapy between the two groups. For BP management, patients at ABC-ICH sites were significantly more likely to receive rapid BP lowering, and door-to-needle times for BP treatment were significantly reduced. However, there was no statistically significant increase in the proportion of patients achieving door-to-target BP lowering. No differences were seen in patient outcomes, such as 7-day mortality or changes in the modified Rankin Scale scores upon discharge compared with non-ABC-ICH sites.

The purpose of this paper is to present findings from the qualitative research study of the North of England scale-up; this aimed to explore which aspects of the implementation strategy and which contextual factors influenced delivery of the ABC-ICH care bundle. Previous researchers have advocated for the incorporation of qualitative methods into quality improvement projects to enable better understanding of outcomes and to provide explanation of the mechanisms of change involved.^12^ Qualitative research helps to understand how and why the implementation of interventions succeed or fail^13^ and is particularly important to capture contextual variation when scaling up interventions across different settings.^14^

## Methods

We report our methods using the Standards for Reporting Qualitative Research checklist.^15^

### Design

We used a mix of qualitative methods. This included semistructured interviews at two time points, non-participant observation of regional meetings and analysis of project documents.

### Conceptual framework

We used the integrated-Promoting Action on Research Implementation in Health Services (i-PARIHS) framework^16^ to guide the research. Developed over the last 20 years, i-PARIHS is a widely recognised theoretical implementation framework^17^ and was chosen for its emphasis on the dynamic interaction between implementation influences and its focus on facilitation as a key ingredient for success.^18^ This aligned with our findings from the previous scale-up of the care bundle in Greater Manchester.^11^ i-PARIHS identifies the following four key factors driving implementation:

*Innovation:* we examined how the care bundle, app and dashboard were understood by users and integrated into practice.
*Context*: we examined how implementation of the care bundle varied across different site contexts.
*Recipients:* we examined how the role of different actors supported implementation at both the individual and collective level.
*Facilitation:* we examined what facilitation resources and strategies were required to support implementation.

### Sampling and recruitment

As facilitation is a key construct of the i-PARIHS framework, we aimed to speak to regional leads responsible for implementing the bundle across their region, and at least one member of the implementation team at the hospitals implementing the ABC-ICH care bundle (in particular, the person leading implementation in the local context). We also wanted to speak to clinicians using the care bundle in the management of patients with ICH, to capture views of the *recipients* of facilitation, namely delivery staff affected by, and influencing implementation of, the care bundle at the organisational level.

### Data collection

All data collection took place between April 2022 and April 2024 and all interviews and observations were undertaken by LB, an experienced qualitative researcher with expertise in conducting process evaluations. We developed a theoretically informed topic guide to guide the interviews (see online supplemental information 2). 30 key stakeholders across six regions in the North of England took part in 45 online interviews. 29 first interviews were conducted (two key stakeholders interviewed together at first interview) and 16 follow-up interviews were conducted approximately 6 months later. Key stakeholders included the six regional leads (five of which were medical consultants and one was a nurse consultant), 15 nurse leads, four consultant leads and five clinicians (nurses) delivering the bundle (see table 1). First interviews lasted for an average of 41 min and follow-up interviews lasted for an average of 30 min. We conducted six observations of regional meetings (lasting between 90 min and 180 min). Handwritten notes were taken during observation and typed up; these were structured around the data presented and discussions held during meetings. We collected nine project documents.

**Table 1:** Participants’ roles in ABC-ICH care bundle project and spread of interviews across regions

Region	Hospital	Role(s) within ABC-ICH QI project	Follow-up interview?
1		Regional lead	✓
	A	Consultant lead	✓
		Nurse lead 1 Nurse lead 2 (interviewed together)	
	B	Consultant lead	✓
2	C	Regional lead (+ consultant lead role)	✓
		Nurse lead	
	D	Nurse lead	✓
		Clinician delivering bundle (nurse)	
	E	Nurse lead	
3	F	Regional lead (+ consultant lead role)	
4		Regional lead	✓
	G	Consultant lead	✓
		Nurse lead	✓
	H	Consultant lead	✓
	I	Nurse lead 1	✓
		Nurse lead 2	✓
		Clinician delivering bundle (nurse)	
5	J	Regional lead (+ nurse lead 1 role)	
		Nurse lead 2	✓
		Clinician delivering bundle (nurse)	
	K	Nurse lead 1	✓
		Nurse lead 2	
		Clinician delivering bundle (nurse 1)	
		Clinician delivering bundle (nurse 2)	
	L	Nurse lead 1	✓
		Nurse lead 2	
6	M	Regional lead (+ consultant lead role)	✓
		Nurse lead	
	N	Nurse lead	✓

QI, quality improvement.

### Data analysis

Interviews were digitally audio recorded, transcribed and anonymised. Data were entered into NVivo 12, a qualitative software program to help manage qualitative data.^19^ We analysed data using the constant comparative method^20^ to enable an iterative mix of inductive and deductive analysis. Data analysis was guided by the i-PARIHS framework, allowing us to draw on established evidence of implementation effectiveness while remaining receptive to emergent insights. LB undertook initial coding of the data, using an adapted version of the coding framework informed by i-PARIHS, developed by Ritchie and colleagues.^18^ Initial codes were refined by LB and PW, an expert in implementation science, to produce a matrix of summarised data for further analysis. Subsequent theme refinement was undertaken by all authors through regular discussion to develop the overarching themes presented in the paper.

### Patient and public involvement

Patients were involved in the overall design of the ABC-ICH care bundle North of England scale-up and were involved in developing materials to promote the quality improvement (QI) project; however, they were not involved in the design of the qualitative process evaluation. Two ICH survivors were invited onto the study’s steering committee.

## Findings

Notably, several unanticipated delays significantly affected planned roll-out and subsequently affected the timing of data collection for the qualitative study. Table 2 provides a summary of implementation across regions. A key delay was due to the difficulties experienced with approving the app and dashboard for use in National Health Service (NHS) Trusts. This was due to a combination of the extensive and varying regulatory and governance documentation required by NHS Trusts, coupled with slow responses from both the software manufacturer and NHS Trusts.

**Table 2:** Summary of implementation across regions

Region	Proportion of planned sites formally implementing the bundle	Summary of implementation across region
1	75%	Formal bundle launch delayed across most sites due to difficulty in organising DPIA approvals (two sites were still in the process of arranging these when app was discontinued). However, ICH protocols changed prior to formal launch (eg, one site changed protocol for BP management). One site launched care bundle >6 months ahead as chose not to implement app. One site did not engage with the ABC-ICH QI project due to changes in clinical leadership but may have made some changes to ICH protocols. A major reconfiguration of stroke services across the region coincided with ABC-ICH QI project and was associated with improvement in key stroke measures. Monitoring of process targets was delayed at some sites due to staff turnover among data leads and incomplete entry of data into SSNAP. Some sites had started auditing ICH care at follow-up interviews. One site had incorporated ICH review into stroke governance meetings.
2	80%	Dual facilitator role: regional/consultant lead role. Formal bundle launch delayed across all sites due to difficulty in organising DPIA approvals (sites were still in the process of arranging these when the app was discontinued). Variable engagement across implementation teams. In some sites, nurse leads took on most implementation work, while consultant leads were less involved throughout the project. This contributed to delays in protocol changes at one site. Some sites reported that ICH data were not being collected for input into SSNAP and no sites were routinely monitoring process targets. One site had recently commissioned a junior doctor to conduct an audit of DTN time for anticoagulant reversal at follow-up interview.
3	33%	Dual facilitator regional/consultant lead role. Regional lead reported challenges with supporting implementation at other sites due to disengagement with project and prolonged DPIA delays over which regional lead had no authority. One site formally withdrew from the ABC-ICH project before implementation, although local ICH protocols may have been revised. One site implemented the ABC-ICH app before it was discontinued, but the app was rarely used in real time. The consultant lead intended to use dashboard data to monitor and feedback process targets to staff; however, limited app use resulted in insufficient data, and the dashboard was not considered user-friendly. Monitoring process targets was deprioritised due to competing clinical demands.
4	100%	Regional lead’s ISDN clinical lead’s role supported implementation across the region due to strategic priorities aligning. Implementation in one site delayed >1 year due to workforce pressures. Two sites used the ABC-ICH app before discontinuation but the app was rarely used in real time at one site and technical issues with dashboard prevented both sites from using data to support monitoring of process targets at follow-up. Attempts to include junior doctors in audit to review early progress at one site were impeded by staffing issues. Future plans included an audit to assess progress at both sites. One site was considering incorporating ICH reviews into stroke governance meetings.
5	50%	Dual facilitator role: regional lead/co-nurse lead role. Formal bundle launch delayed across all sites due to difficulty in organising DPIA approvals, but some sites had started to implement ICH protocol changes prior to formal launch. One site did not engage with the ABC-ICH QI project, due to staffing issues, but may have made some changes to ICH protocols. Some sites did not have a full complement of staff to fulfil ABC-ICH QI project roles, with implementation being led by nurse leads across the majority of sites, with minimal consultant lead input. This led to delays in agreeing ICH protocol changes in some sites. None of the sites were routinely monitoring process targets but one site was considering incorporating ICH reviews into stroke governance meetings.
6	100%	Dual facilitator role: regional lead/consultant lead role. Official bundle launch delayed at one site due to staff changes and ward reconfiguration. Engagement from wider implementation team was variable at this site with the nurse lead undertaking the majority of project-related tasks. Monitoring process targets was deprioritised due to competing clinical demands and at follow-up interview, ICH data were still not being entered into SSNAP by the data lead, as this was not compulsory. Progress targets were monitored proactively at another site with the inclusion of ICH review within stroke governance meetings.

BP, blood pressure; DPIA, Data Protection Impact Assessment; DTN, door-to-needle; ICH, intracerebral haemorrhage; ISDN, Integrated Stroke Delivery Network; QI, quality improvement; SSNAP, Sentinel Stroke National Audit Programme.

Other barriers delaying bundle launch included HASU-level staff shortages, extra pressures placed on staff due to COVID recovery, national doctors strikes and ward restructures. As a result, individual HASUs within a region often implemented the bundle at different times (or in some cases, not at all), leading to a staggered and variable roll-out within and across regions. This meant that regional induction meetings often happened several months before the bundle launched in some hospitals and contributed to regional follow-up meetings not occurring within planned timeframes. This led to limited engagement from some HASU implementation teams.

In addition, in July 2023, due to the difficulties experienced with gaining regulatory approvals and app usability issues, a decision was taken to discontinue the app and dashboard. These were replaced with the Sentinel Stroke National Audit Programme (SSNAP) which was adapted to include an optional section for participating ABC-ICH sites to complete. The following qualitative findings are presented within the context outlined above, providing insight into the factors affecting implementation of the ABC-ICH care bundle at scale. Table 3 provides direct quotes to support the interpretation of findings.

**Table 3:** Quotes to support themes

Theme	Quote
Theme 1: perceived clarity, alignment with clinician values, and flexibility act as facilitators to uptake	I think previously there’s not really been much that we can give to patients that have had a bleed on the brain, so it’s sort of like a sit and wait scenario. Whereas now, you actually feel like you’re doing something to maybe help the bleed, to stem it and control it, and try to improve outcomes. **Clinician (nurse) delivering care bundle, hospital I, region 4, one-time interview**. Well, so we had started to look at those components… in the ABC-ICH, obviously not necessarily looking at it from a bundle perspective but had been looking at how do we improve anticoagulation reversal how do we improve blood pressure lowering. **Regional lead for region 3, first interview** … the other good thing with the project is that I know [central project team] were quite happy for us to use a local guideline as long as the blood pressure comes down. […] initially, we thought we’ll have a joint guideline … between the two Trusts but…we didn’t feel that at that stage [it] was going to be feasible. **Regional lead for region 6, first interview**.
Theme 2: usability issues and lack of contextual fit act as barriers to sustaining the app support component	I suppose what would be useful is some sort of mobile friendly app which is touchscreen friendly. Because we’re used to that now. Simple things. …[the app] doesn’t do it. You’ll have to press next to go to the next screen and it’s not always clear how to go from A to B to C. **Regional lead 2, region 6, first interview**. The stroke nurses did voice, particularly at night, when there’s less staff around, and they're putting [data] into the app, *and* into the clinical system. And I said, focus on the clinical system, because that’s what’s going to be looked at by everybody else, whereas not everybody will access the app. **Consultant lead, hospital G, region 4, follow-up interview**. Q: Did you actually ever use the app in real time when implementing the bundle? Yes, and I found it really, really tricky. I found it tied me to that patient for longer than I needed to be there. **Clinician 1 (nurse) delivering bundle, hospital K, region 5, one-time interview**.
Theme 3: lack of recipients’ skills and cultural norms act as barriers to integrating the C component of the ABC-ICH care bundle	And then the neurosurgical thing is just such a big culture change. Absolutely enormous culture change that I think, the problem … is, I don't feel like I have that much power to make inroads into it. **Regional lead for region 2, follow-up interview**. Yeah, well, I’d like to be honest with you, the C part we don’t focus on too much because…some of my colleagues are of the impression that they would refer to neurosurgery… though they know that they wouldn’t do anything. I mean, just as a… [they] want a second opinion. **Regional lead for region 6, follow-up interview**. Q: Do you feel then that using the ABC-ICH bundle is becoming routine care for patients with ICH at your hospital? Most of it is. The reason I say most of it is, is because from the ABC-ICH bundle, the bundle, kind of, guided which patients, you know, the last part of it, consultation with the neurosurgeons. Not everybody needs to be referred to the neurosurgeons, but that part I think still we are struggling. Every bleed gets referred to neurosurgeons, and like, you don’t need to refer this one. **Clinician 2 (nurse) delivering care bundle, hospital K, region 5, one time interview**.
Theme 4: lack of routine monitoring and feedback mechanisms act as barriers to implementation and undertaking improvement cycles	I think what we struggled with was getting people to see it or do it as a PDSA [cycle] rather than just a change to their pathway. […] certainly at [one local hospital] I know that they have done a cycle of review and come up with some new recommendations. **Regional lead for region 1, follow-up interview**. And yeah, I think our biggest issue probably is data collection to see whether we’re actually achieving [process targets] just now. **Nurse lead, hospital D, region 2, follow-up interview**. I’ve not had any feedback in terms of whether it’s improved outcomes, but I know [nurse lead]’s doing some work in evaluating it recently, so we might hear some stuff shortly. […] But yeah, I’d just like to know whether it’s doing its job really. Whether it’s worth continuing and stuff. **Clinician (nurse) delivering bundle, hospital I, region 4, one-time interview**.
Theme 5: how contextual enablers and barriers affect facilitation	the time I’m spending on this project is recognised as contributing towards my [ISDN clinical lead] role…if I perceived it as a conflict or a drain on my time or something that wasn’t relevant to my other major paid roles, I think it would’ve been more difficult for me to sustain the intensity and, I guess, the passion towards making this project successful. **Regional Lead for region 4, first interview**. …what I tried to do, was set up regular meetings to occur across all three [Trusts], [but] on most occasions, no one from [one HASU] turned up… [there was] more engagement from a [other HASU] perspective in terms of attendance, but…none of them have attended at the same time… I know that it’s been discussed at the ISDN level, but I don’t know [as] I’m not party to any of those discussions that have taken place at that level. **Regional lead for region 3, first interview**. I think you always need it to have that senior medical input. Because I think when you're going to… the haematologists and asking them if you can create a protocol about anticoagulation reversal, it’s the consultant haematologist that are going to reply, so they're expecting you to go as a consultant stroke physician. […] And interestingly, that’s still the area where the [HASU name] has struggled in terms of nailing down protocol… They've been great as a specialist nurse team, and to be fair their specialist nurses have pushed for protocols, but that is still the area where they've struggled. **Regional lead for region 2, follow-up interview**.

HASU, hyperacute stroke unit; ICH, intracerebral haemorrhage; ISDN, Integrated Stroke Delivery Network; PDSA, plan, do, study, act.

### Theme 1: perceived clarity, alignment with clinician values and flexibility act as facilitators to uptake

The clarity with which the innovation was understood was a key success to the ABC-ICH care bundle being integrated into care, as it provided clinicians with a clear and systematic pathway to deliver ICH care, something which they previously lacked.

A strong theme throughout interviews was that before introducing the ABC-ICH care bundle, ICH care was considered the ‘Cinderella’ of stroke services (clinician delivering bundle—nurse 1, region 5, first interview). Respondents reported that ICH care was not prioritised in the same way as ischaemic stroke care, which benefited from having robust policies and pathways already in place. The relative advantage of introducing the ABC-ICH care bundle was that it had raised awareness among clinicians to now aim to treat ICH patients with the same urgency as patients with ischaemic stroke, reducing therapeutic nihilism.

The ABC-ICH care bundle was a good fit with the values and norms of the individuals/teams implementing it. Some regional leads described how the bundle was compatible with the priorities set out within their Integrated Stroke Delivery Networks (ISDNs), a regional partnership bringing together stroke services across the stroke pathway to improve coordination, quality and equity of stroke care. Some reported having already made attempts to start improving care components described in the ABC-ICH care bundle prior to implementation. In addition, several respondents described contacting the central project team in Manchester for advice to adapt the bundle to their local context, after hearing about it at conferences or reading academic papers, prior to taking part in the North of England scale-up.

Another key strength of the ABC-ICH bundle was its flexibility: although a central set of process targets was defined, participating sites were encouraged to adapt or develop protocols for anticoagulant reversal, intensive BP lowering and care pathway for neurosurgical referral that aligned with their local hospital policy context, supported by examples from the central project team. Most hospitals within and across regions had different standard operating procedures or policies for managing reversal of anticoagulant medication and BP lowering (eg, by choosing different first-line drugs). Attempting to standardise protocols across hospitals within a region was considered too complex to organise within the project timeframe; hence, the flexibility of the care bundle was considered necessary for successful launch.

### Theme 2: usability issues and lack of contextual fit act as barriers to sustaining the app support component

As previously explained, a key barrier to launching the ABC-ICH care bundle was the delay in gaining the necessary hospital approvals for the use of the app and dashboard. Additionally, once the app was in use, several usability issues and lack of contextual fit were identified. Due to these challenges, the app and dashboard were ultimately discontinued. The intended purpose of the app was to guide delivery of the ABC-ICH care bundle in ‘real time’ which in turn populated the dashboard with data, to be used by local implementation teams to identify whether process targets were being met.

In the main, nurses were tasked with inputting data into the app, given they were most likely to initiate delivery of the ABC-ICH care bundle. However, it was reported that nurses did not often use the app in ‘real time’ as it was considered an extra burden on top of an already busy workload. The app was not considered to be user friendly nor an aid to deliver bundle components. Lack of computers in some ward areas and poor Wi-Fi connections in some hospitals also impeded its use; reliable internet access was required because the app’s core functionality was delivered through a web-based platform. Some local implementation teams purchased iPads to encourage app usage, but governance restrictions in one hospital required separate iPads to be used for different projects, leading to confusion over which device to use, further reducing engagement with the app. Inputting data into the app also created duplication of data entry, and clinicians prioritised inputting data into patients’ medical records, further limiting its use in practice.

### Theme 3: lack of recipients’ skills and organisational cultural norms act as barriers to integrating the C component of the ABC-ICH care bundle

Most regions had collaborated closely with their neurosurgical leads to develop protocols outlining criteria to refer ICH patients to neurosurgery, which were formally agreed upon by their partnering hospital Trusts. Despite this, most regional leads and local implementation team members indicated that referral criteria were not being adhered to, with many reporting that ICH patients continued to be referred to neurosurgery, regardless of eligibility. Some suggested that during working hours, when senior stroke consultants were more likely to be present, the stroke team would follow the agreed referral criteria. In contrast, ICH patients under the care of more junior doctors, nurses or those in the ED were more likely to be routinely referred, irrespective of the criteria. This was attributed to department cultural norms of routinely referring all ICH patients for neurosurgical opinion—a practice that was considered difficult to change quickly. Some regional leads expressed feeling powerless to influence this practice.

In addition, lack of skills and knowledge among clinicians in calculating haematoma volume was reported to further impede their ability to apply the referral criteria. Accurate haematoma volume calculation was a requirement for using the neurosurgical referral guidelines; therefore, many defaulted to blanket referral due to lacking confidence in their calculations. Despite local implementation teams identifying this as a gap in skills, there was no evidence of plans to address this. Instead, implementation efforts were prioritised to other components of the care bundle where local implementation teams felt that there was greater opportunity to influence practice and achieve meaningful change.

### Theme 4: lack of routine monitoring and feedback mechanisms act as barriers to implementation and undertaking improvement cycles

Most sites struggled to collect data and monitor process targets following implementation of the ABC-ICH bundle. At both the initial and follow-up interviews, respondents commonly reported that local implementation teams were not routinely monitoring whether process targets were being met. As a result, few sites had undertaken PDSA cycles, which limited their ability to drive further improvement in ICH care.

Lack of monitoring was due to several overlapping factors. In some cases, before discontinuation of the app and dashboard, some sites had chosen to not use the app, for example, in one hospital this was due to their IT governance lead refusing to support its implementation (due to governance concerns). As previously explained, when the app was in service, it was not being used in real time. This meant that data did not appear in the dashboard as intended, and local implementation teams did not have the time to input data retrospectively. In region 4 (the first to implement the ABC-ICH care bundle), additional technical issues with the app’s dashboard feature meant that even when data were entered, they were not accessible to review. When the app was discontinued, local implementation teams did not consistently use the SSNAP dashboard to monitor progress. Clinicians in local implementation teams were not often given dedicated time by their organisations to undertake the ABC-ICH care bundle QI project and had to undertake project duties alongside their clinical duties—this meant that clinical duties took priority. Some respondents considered that without mandatory national auditing of ICH care, it would remain difficult to monitor delivery of the ABC-ICH care bundle, arguing that it was viewed as less of a clinical priority compared with ischaemic stroke care, which was already subject to national audit.

More encouragingly however, several local implementation teams described plans to start monitoring data more closely in the coming months, with some reporting that they had begun to incorporate ICH management into existing stroke governance meetings. These were meetings where thrombolysis and thrombectomy cases were already discussed, to identify areas for improvement. Others indicated plans to do so in the near future.

### Theme 5: how contextual enablers and barriers affect facilitation

A key factor contributing to the timely launch in one region (region 4) was that the ABC-ICH QI project regional lead also served as a clinical lead for their regional ISDN. These dual roles were considered compatible, as the aims of the ABC-ICH project were compatible with the organisational priorities of the ISDN. This alignment enabled the regional lead to dedicate sufficient time to facilitate implementation across their region. More broadly, regional leads perceived that the formation of ISDNs facilitated collaborative working across HASUs, fostering a culture of cooperation, to the benefit of initiatives like the ABC-ICH QI project.

In contrast, at the time of the first interviews, regional leads in two regions (regions 3 and 6) were struggling to launch the bundle region-wide, with implementation limited to their own hospitals. These regional leads perceived a lack of authority beyond their own Trusts and felt disconnected from ISDN-level discussions intended to encourage wider implementation. In addition, both regional leads in these areas also undertook the consultant lead roles for the ABC-ICH QI project at their own hospitals, effectively performing two roles. This dual responsibility, combined with a perceived lack of influence over regional implementation delays, and limited time to devote to the project, may have led them to focus efforts primarily within their own Trusts rather than across the entire region.

In most hospitals, the presence of nurse leads was a key enabling factor in progressing implementation. Nurse leads often coordinated staff training on the ABC-ICH care bundle and were the point of contact for questions and ongoing support. Unlike the Greater Manchester scale-up, there was no formal launch of the care bundle at participating sites (due to ongoing COVID-19 and other staff pressures). High workloads and staffing pressures sometimes also prevented opportunities for structured training sessions; however, nurse leads mitigated this by providing flexible one-to-one support, including informal bedside training, to ensure nursing staff were familiar with the care bundle. They also acted as the point of contact for questions and implementation challenges.

Not all sites had a full complement of staff to fulfil the implementation team roles outlined by the central project team, and this contributed to variable implementation outcomes. For example, some local implementation teams lacked sustained input from medical consultants. This may have undermined clinical leadership and the perceived legitimacy of the QI project, especially where consultant engagement was expected to drive clinical protocol change. By contrast, hospitals with medical consultant leads described successful relationship-building with haematologists and neurosurgeons, enabling them to influence changes in protocols more easily. Limited involvement from data leads in some hospitals also resulted in inconsistent or delayed data entry into SSNAP, potentially impacting both local monitoring and wider project evaluation.

To illustrate how the five themes above align with the i-PARIHS framework, table 4 outlines where each theme is conceptually situated across the four constructs, identifying how multiple constructs interact to shape implementation.

**Table 4:** Mapping facilitators and barriers within themes to the four constructs of i-PARIHS

Theme	Innovation	Recipients	Context	Facilitation
1. Key facilitators to adoption of A and B parts of care bundle	Clarity of innovation Relative advantage compared with previous practice Flexibility in allowing adaptation to specific protocols			
2. Key barriers to using app support component (led to discontinuation)	App not user-friendly Creates duplication of data entry		Slow response from app company and hospital governance to approve app Lack of computers in ward spaces, poor Wi-Fi and busy workloads impede use	
3. Key barriers to adoption of C part of care bundle		Lack of skills and confidence to calculate blood volumes	Cultural norm to refer all to neurosurgery	No plans to upskill staff
4. Key barriers to driving further improvement in ICH care	Technical issues with app and dashboard (when in use)		No protected time to undertake QI project work Monitoring ICH process targets less of a priority (compared with ischaemic stroke care which is nationally audited)	Local implementation teams not routinely monitoring process target data in SSNAP (after app and dashboard discontinued)
4. Key facilitator to driving future improvement in ICH care				Plans to include ICH management in existing stroke governance meetings
5. Key facilitators to effective facilitation			Regional lead is also clinical lead for ISDN ABC-ICH QI project’s aims aligned with ISDN’s organisational priorities ISDN’s facilitate collaboration across HASUs	Local consultant leads building relationships with key stakeholders enable protocol change Nurse leads providing training and ongoing support to staff
5. Key barriers to effective facilitation			Some regional leads lacked authority beyond own hospital to enact change	Dual implementation team roles (regional lead+local consultant lead) may overburden clinicians Some local implementation teams unable to fulfil all roles

HASUs, hyperacute stroke units; ICH, intracerebral haemorrhage; ISDN, Integrated Stroke Delivery Network; QI, quality improvement; SSNAP, Sentinel Stroke National Audit Programme.

## Discussion

In the present study, we identified several key factors for influencing the uptake and sustainability of the ABC-ICH care bundle when scaling up across six regions in the North of England.

Key facilitators in the adoption of the ABC-ICH care bundle included the clarity with which the care bundle was understood, its perceived relative advantage compared with current clinical practice and its compatibility with staff values. Findings from this study are consistent with findings from our previous study which identified that the ABC-ICH care bundle was compatible with clinicians’ values and acted to provide clear guidance to deliver ICH care.^11^ Introduction of the care bundle encouraged staff to treat ICH patients with the same immediacy and priority as ischaemic stroke patients, and the bundle served to counteract the therapeutic nihilism reported in earlier research.^3 4^ This cultural shift appears to have encouraged a more proactive and standardised approach to ICH care. This supports the wider implementation science literature which highlights that innovations are more likely to be adopted into practice when they have a clear and unambiguous advantage over current practice and are compatible with the values and beliefs of the staff expected to deliver them.^21–23^

In this study, we found that allowing adaptation of the specific protocols (ie, rapid anticoagulant reversal, intensive BP lowering and appropriate and prompt referral to neurosurgery) to fit local context supported local implementation. Previous literature highlights the importance of ‘plasticity’—the extent to which components of an intervention can be moulded to fit their context.^24^ Maintaining fidelity to the *function* of an intervention (in this case, the process targets) rather than to the *form* (such as imposing the use of a specific first-line drug in rapid BP lowering) is considered important for improving effectiveness and sustainability.^25 26^

In the present study, we found that the C component of the ABC-ICH care bundle was not widely adopted beyond senior stroke clinician teams, due to a lack of staff knowledge in calculating haematoma volume (a necessary requirement to make a decision to refer to neurosurgery), and prevailing cultural norms where all patients were referred for neurosurgical review. This was consistent with the findings from the previous study, where we found that clinicians continued to make unnecessary referrals to neurosurgery despite the care pathway being in place.^11^ Across both studies, we identified that most staff outside of core stroke teams lacked the competence and confidence to calculate blood volumes, highlighting a clear deficit in recipients’ knowledge, skills and self-efficacy. Despite highlighting this barrier during the North of England scale-up induction meetings, we argue that limited capacity in local implementation teams resulted in resources being directed towards areas where they perceived change to be more achievable. However, this highlights the need for targeted staff training and guided practice to build confidence for staff to adhere to the new care pathway.

A consistent theme identified was the central role that facilitation played in implementation. Local facilitators were effective in processes such as planning for implementation, fostering relationships with key stakeholders and providing support to staff. We also found that meso-level contextual factors, such as the recently formed ISDNs, served to improve collaborative working across hospital Trusts, which in turn supported facilitation processes for the ABC-ICH project. This is consistent with previous research which identifies the relational, organisational and knowledge mobilisation mechanisms through which facilitation influences implementation.^27^ However, local contextual issues such as competing clinical priorities and lack of protected time to undertake QI project activities meant that local implementation teams were less effective in monitoring data and undertaking PDSA cycles, limiting their ability to gain further improvements in ICH care. Similar barriers to implementation success, including lack of organisational support, not prioritising QI activities and insufficient time and resources, were identified in a systematic review assessing the effectiveness of QI collaboratives to improve stroke care.^28^ Previous research has identified monitoring and evaluation as a key step in facilitation for implementation success.^29^ Facilitation was also constrained when regional leads lacked authority beyond their own hospital Trusts to influence change, and where gaps in implementation team roles reduced local capacity to support implementation. Using the i-PARIHS framework has shown the complex interplay between facilitation as the ‘active ingredient’ and the core constructs of innovation design, recipient engagement and contextual readiness.^30^ Our findings highlight that facilitation alone is insufficient to drive change.

### Limitations

Our findings need to be considered in light of the recruitment and data collection issues we experienced during the study. We aimed to recruit a mix of key informants in various roles across the six regions. We successfully recruited most clinicians who were leading local implementation at each hospital; however, we were not successful in recruiting any data leads into the study—those that replied to decline participation reported they had little engagement with the QI project and did not have anything meaningful to contribute. In addition, we struggled to recruit medical clinicians delivering the bundle outside of local implementation teams and were unable to conduct planned follow-up interviews with some local implementation team leads and two regional leads as intended. When respondents declined follow-up interviews, they cited lack of capacity, reflecting the high workload and staffing issues present within the NHS at the time of data collection. Therefore, our findings may not fully capture all clinicians’ perspectives, as non-responders may have had different experiences with the ABC-ICH care bundle.

We conducted non-participant observation at six regional meetings which all took place online. These produced limited data as they did not generate rich discussion, compared with earlier in-person meetings that occurred in the Greater Manchester scale-up, as previously reported.^11^ Consequently, observation data were mainly used for contextual background rather than as a key source of analytical insight.

### Implications for practice

Despite the limitations outlined above, these findings have implications for practice. The quantitative evaluation indicates that the ABC-ICH care bundle is suitable for large-scale national implementation. However, we suggest the following refinements before full national roll-out:

1. *Strengthen monitoring and feedback mechanisms*. Although the ABC-ICH care bundle is well liked and aligns with ISDN priorities, local implementation teams report challenges in having dedicated time to monitor progress. As monitoring becomes more formalised (eg, via SSNAP), hospitals may give greater priority to improving ICH process measures.
2. *Collaborate with ISDNs for regional coordination*. We recommend partnering with ISDNs to support national implementation, by helping to embed the care bundle, share learning and address barriers to implementation. Future national roll-out should include dedicated funding for regional leads to provide protected time for coordinating and supporting implementation across their regions.
3. *Limit the number of roles clinicians take on*. For organisations implementing the ABC-ICH care bundle as part of a wider QI project, we recommend limiting each clinician to one implementation team role to reduce being overburdened and to maintain engagement.
4. *Provide targeted staff training in blood volume calculation*. To support consistent adherence to the new care pathway, relevant clinicians should receive dedicated training in blood volume calculation. This will build confidence and help ensure the care pathway is implemented reliably across teams.

## Supplementary material

10.1136/bmjoq-2025-004099 online supplemental file 1

10.1136/bmjoq-2025-004099 online supplemental file 2

## Data Availability

Data are available upon reasonable request.
