# Supplementary material for: Rollout of a care bundle for intracerebral haemorrhage across the North of England: a qualitative study
Source: BMJ Open Qual. 2026 Aug 26;15(3):e004099. doi: 10.1136/bmjoq-2025-004099 (PMC13536107; doi:10.1136/bmjoq-2025-004099)
Supplement: Supplementary file 1 [file bmjoq-15-3-s001.pdf]

Outline of implementation team roles provided to teams ahead of launch

<u>HASU Implementation Team</u>

A Stroke Consultant, Stroke Nurse and Data Lead will be identified to form the core implementation team at each HASU. The data lead role will typically be responsible for entering SSNAP data for the service. Other team members (including trainees keen to gain QI experience) may be added at the team's discretion. It may be useful to have associate members from outside of the Stroke Department, depending on local context. For example, an associate team member from the Emergency Department may be useful if ED staff play a key role in delivering hyperacute ICH care at the HASU. The following will be the expectations of all members of the core team:

• Attend a regional induction meeting (1 day) and participate in regional meetings 3 & 6 months after launch (half day each).

• Participate in regular (every 1-2 weeks), short (< 30 min) HASU implementation team meetings during the project period (9 months: starting from regional induction and finishing 6 months after bundle launch).

• During the set-up period (3 months: from regional induction to bundle launch), key tasks will be:

  o Modifying ABC protocols (templates will be provided) for local use and getting approval, as per local clinical governance arrangements

  o Engaging key stakeholders in other hospital departments (Haematology, Critical Care, Emergency Department)

  o Arrange and deliver a local launch meeting (a slide set will be provided)

  o Assisting MDSAS (ABC-ICH app developer) with getting ABC-ICH app and dashboard set-up at the HASU

  o Preparing to deliver training to clinicians managing acute ICH patients (materials to be provided)

• After the bundle launch date (6-month period) key tasks will be:

  o Train any new staff arriving in this period

  o Review process data (through ABC-ICH dashboard) and identify any possible changes that may lead to improvement

Outline of implementation team roles provided to teams ahead of launch

o Test changes as needed, using PDSA cycles

Individual HASU team member responsibilities

The following are roles that are specific to each team member:

Stroke Consultant:

• Provide leadership of team and ensure the above aims are being met. Leadership of the team need not necessarily be the consultant and this could be another team member, as agreed locally

• Engage consultants in both Stroke and other Departments to seek their support for the project

• Engage senior leaders in the NHS Trust to facilitate removal of any barriers encountered

Stroke Nurse:

• Ensure nursing aspects of protocols are implemented and training is provided to existing and new nursing staff

Data lead:

• Improve completeness and quality of SSNAP audit data, if required

• Send postal mRS questionnaires (a project template questionnaire and smRSq form will be provided for local adoption) and call non-responders. This may be delegated to a member of the clinical team, if needed.

• Add cases retrospectively to ABC-ICH database / dashboard if app not used acutely

• Access the ABC-ICH dashboard during team meetings to present progress to team

Regional leadership team

At a regional level, the project will be led by a Stroke Clinician (Stroke Physician or Stroke Nurse). A Neurosurgeon will be identified to support the project regionally. The following are the expectations for each role:

Regional Clinical Project Lead:

Outline of implementation team roles provided to teams ahead of launch

- Promote and provide leadership for the project regionally, with support from the Central Leadership Team.
- Participate in monthly Project Management Group teleconferences throughout the course of the project (Apr 2021 to Sep 2022), along with all other Stroke Regional Leads, the Central Leadership team and representation from the regional AHSNs.
- Help to organise and facilitate the three regional meetings (induction meeting, 3- & 6-month meetings)
- Liaise with local AHSN and ISDN, as needed
- Provide support to HASU teams, with advice and support from Central Leadership Team
- Lead development and approvals of any regional pathway changes directly relating to the ABC-ICH project

Neurosurgical Regional Lead:

- Participate in regional meetings (induction meeting and 3- & 6-month meetings)
- Support Stroke Regional Lead by promoting the project with Neurosurgical colleagues, helping to develop regional pathway changes and ensuring good communication between Neurosurgery and Stroke
- Support Stroke Regional Lead in liaising with the Critical Care team at the Neurosciences Centre, especially if Stroke Regional Lead is not based at the Neurosciences Centre

<u>Central Leadership team</u>

Central Project Lead

- Provide overall leadership and direction for the ABC-ICH project across the North of England
- Develop all project materials (website, protocols, training materials, slide sets, videos)
- Lead regional meetings in collaboration with Regional Project Clinical Lead
- Liaise with SSNAP central team to develop reports for meetings

Outline of implementation team roles provided to teams ahead of launch

• Liaise (along with Overall Project Manager and HP) with Project Steering Group to guide and steer overall project. The Project Steering Group will be comprised of independent expert clinicians, patient representatives and representation from NHS England and SSNAP and will meet by teleconference three times during the course of the scale up.
• Lead research evaluation of ABC-ICH

Central Project Manager
• Day-to-day running of the quality improvement project, ensuring actions are completed to time and target.
• Responsive and regular communication with 6 regional leads and 20-25 hospital teams taking part in the project, to ensure effective and efficient delivery of the project, including handling of ad hoc queries, a regular newsletter, development and maintenance of the project website and management of the project Twitter feed.
• Support communication with participating NHS Trusts (including contact with Trust Executives), Integrated Stroke Delivery Networks in each region and local AHSNs.
• Work with MDSAS to finalise the ABC-ICH app and dashboard and associated materials. Support implementation of app and dashboard in 20-25 participating hospitals.
• Ensure all project materials (including protocols, training materials, slide sets, videos) are prepared and available prior to the first regional induction meeting (August 2021), liaising with video production company and MDSAS, as required.
• Help organise, prepare, and deliver 6 regional induction meetings (1 day each) as well as 3-months & 6-month post-launch meetings (12 in total, half day each). Liaise with SSNAP to ensure data reports from each hospital are available for review at each meeting as part of the slide set.
• Work with data leads at each hospital to ensure data entered in to SSNAP are complete and accurate, including support to improve collection and completeness of

Outline of implementation team roles provided to teams ahead of launch

6-month mRS.

• Monitor progress at sites and maintain the ABC-ICH dashboard. Ensure that learning is shared between sites, including regularly highlighting successful PDSA cycles to other sites.

• Support health economics PDRA working on the Stroke Association ABC-ICH Research Project, including preparation of applications for ethics approval, and supporting export and linking of data from SSNAP via NHS Digital to HES and ONS data.

• Support qualitative PDRA working on the Stroke Association ABC-ICH Research Project, including support with ethics applications, access to hospital sites and support to arrange interviews and non-participant observation.

• Support set up and running of the Project Management Group (to meet monthly from Apr 2021) and Project Steering Committee (to meet on 3 occasions during project), including preparation of agenda, reports, and other materials.

• Support dissemination of findings via conferences, academic papers, social media, plain English reports and patient and public forums, such as the UK Stroke Assembly.

• Support liaison with NHSE and local commissioners to prepare for national roll-out of bundle, on successful completion of the Northern Scale-up.

Central Neurosurgical Lead

• Work with Neurosurgical Regional Leads to support project in each region

• Attend all regional induction meetings and lead Neurosurgical aspects of these meetings
