# Supplementary material for: Rollout of a care bundle for intracerebral haemorrhage across the North of England: a qualitative study
Source: BMJ Open Qual. 2026 Aug 26;15(3):e004099. doi: 10.1136/bmjoq-2025-004099 (PMC13536107; doi:10.1136/bmjoq-2025-004099)
Supplement: Supplementary file 2 [file bmjoq-15-3-s002.docx]

**Professional role:**

What is their current professional role?

How long have they worked in their current role?

**Role in ABC-ICH bundle implementation**

What is their role in implementing the ABC-ICH bundle?

How did they hear about proposals to implement the bundle?

What were their initial thoughts on the ABC-ICH bundle?

How do they feel it differs to how they managed ICH patients before?

**Elicit how implementation is being managed**

Who is leading the implementation of the ABC-ICH bundle?

What stage of implementation are they at?

How do people work together to implement ABC-ICH bundle?

Number of clinical staff expected to use the bundle.

What factors have influenced the implementation of ABC-ICH bundle?

**What support has been given to implement ABC-ICH bundle?**

E.g. training for staff

How was training delivered (group size/time taken to deliver)

How is training fitted around clinical duties?

On-going support

Have any groups been formed to oversee implementation?

Any associated costs with implementing the bundle?

Describe what they were

**How are they monitoring implementation of ABC-ICH bundle**

How often are they monitoring performance of ABC-ICH bundle?

What mechanisms are in place for providing feedback on ABC-ICH bundle to staff?

What mechanisms are in place to improve the ABC-ICH bundle? (i.e. through use of ‘real time data’ performance)

How are staff encouraged to use ABC-ICH bundle?

**What challenges. successes have they encountered implementing the ABC-ICH bundle**

How were these addressed?

Do they feel the ABC-ICH bundle has been sustained into routine practice?

If not, why do they feel it hasn’t worked? /if yes, what factors do they think enabled implementation

How do they feel they could have improved the way ABC-ICH bundle was implemented?

Does bundle take longer to implement/more resource than previous routine care? Does this change as clinicians get used to using the bundle?

Ask them to describe positives of ABC-ICH bundle

**Perceived outcomes of implementation of ABC-ICH bundle**

What do their colleagues think about the implementation of the bundle

Is there any resistance to using it?/why?

How do they think bundle could be improved?

How they feel it has improved care for patients

**How implementation of the bundle changed their working practices**

How has it changed their interaction with multi-disciplinary team members?

[How] do they receive feedback on how it has improved patient care?

How would they like to receive feedback on how/if ABC-ICH bundle improves patient care

**Final comments**

How have they found the experience of being involved in the implementation of the ABC-ICH bundle?

What would they do differently next time?

Do they have anything else they would like to say?
